# Regulated Expression of a Cytokinin Biosynthesis Gene *IPT* Delays Leaf Senescence and Improves Yield under Rainfed and Irrigated Conditions in Canola (*Brassica napus* L.)

**DOI:** 10.1371/journal.pone.0116349

**Published:** 2015-01-20

**Authors:** Surya Kant, David Burch, Pieter Badenhorst, Rajasekaran Palanisamy, John Mason, German Spangenberg

**Affiliations:** 1 Department of Environment and Primary Industries, Biosciences Research Division, the Grains Innovation Park, Horsham, Victoria, Australia; 2 Department of Environment and Primary Industries, Biosciences Research Division, Hamilton, Victoria, Australia; 3 Department of Environment and Primary Industries, Biosciences Research Division, AgriBio, Centre for AgriBioscience, Bundoora, Victoria, Australia; 4 La Trobe University, Bundoora, Victoria, Australia; Leibniz Institute of Plant Biochemistry, GERMANY

## Abstract

Delay of leaf senescence through genetic modification can potentially improve crop yield, through maintenance of photosynthetically active leaves for a longer period. Plant growth hormones such as cytokinin regulate and delay leaf senescence. Here, the structural gene (*IPT*) encoding the cytokinin biosynthetic enzyme isopentenyltransferase was fused to a functionally active fragment of the *AtMYB32* promoter and was transformed into canola plants. Expression of the *AtMYB32xs::IPT* gene cassette delayed the leaf senescence in transgenic plants grown under controlled environment conditions and field experiments conducted for a single season at two geographic locations. The transgenic canola plants retained higher chlorophyll levels for an extended period and produced significantly higher seed yield with similar growth and phenology compared to wild type and null control plants under rainfed and irrigated treatments. The yield increase in transgenic plants was in the range of 16% to 23% and 7% to 16% under rainfed and irrigated conditions, respectively, compared to control plants. Most of the seed quality parameters in transgenic plants were similar, and with elevated oleic acid content in all transgenic lines and higher oil content and lower glucosinolate content in one specific transgenic line as compared to control plants. The results suggest that by delaying leaf senescence using the *AtMYB32xs::IPT* technology, productivity in crop plants can be improved under water stress and well-watered conditions.

## Introduction

Senescence is a highly regulated degradative process of plant cellular and tissue structures. Delaying the senescence of source tissues (mainly leaves) would permit greater capture of sunlight energy for an extended period, allowing transformation into photosynthates which contribute to improved plant growth and enhanced seed yield. Additionally, delayed senescence would allow source tissues to degenerate slowly, so that the stored photosynthates, metabolites, proteins and nutrients can be systematically released and dispatched to corresponding sink tissues. The benefits of delayed leaf senescence include better maintenance of photosynthetic rate, increased plant biomass, higher nitrate influx, increased post-harvest life in flowers, enhanced drought tolerance and higher seed yield [1–6].

The plant growth hormone cytokinin plays a vital role in promotion of cell division, growth and differentiation, and influences several developmental and physiological aspects in plants including seed germination, apical dominance, flowering time, flower and fruit development and leaf senescence [7–9]. The role of cytokinins in delaying leaf senescence has been reported for several plant species [1–4,10–12]. In these studies, the endogenous cytokinin level was enhanced by the expression of an *Isopentenyltransferase* (*IPT*) gene isolated from *Agrobacterium tumefaciens*, as the IPT enzyme catalyses the rate-limiting step in cytokinin synthesis.

Regulated expression of the *IPT* gene is crucial for obtaining in gaining the beneficial effects from increased cytokinin levels. Early research focused on the use of strong constitutive promoters [13]. This resulted in abnormally high endogenous cytokinin levels and constrained the establishment of transgenic plants due to inhibition of normal root system formation. Additionally, the established transgenic plants exhibited morphological and physiological abnormalities [14,15]. Inducible heat shock-responsive promoters were also used [16,17]. However, such plants exhibited poor root growth and enhanced auxiliary bud growth due to ‘leaky’ expression of the inducible promoters under non-inductive conditions. Later, it was realised that a minimal increase in *IPT* expression is sufficient for the beneficial effects on plant development [18].

Gan and Amasino [4] first used the senescence-specific promoter, *SAG12* fused with the *IPT* gene to explore the role of cytokinins in delaying leaf senescence and reported higher biomass and yield in transgenic tobacco plants. The *SAG12* promoter was subsequently used for such experiments in various plant species [2,5,6,19–21]. Notably, several beneficial effects were observed in these reports, such as increased nitrate flux, higher nitrate reductase activity and delayed leaf senescence. However, developmental abnormalities such as nutrient deficiency, delayed flowering, and lower seedling establishment were also observed in some transgenic plants [5,21,22].

Recently, a stress- and maturation-induced promoter from the senescence associated receptor protein kinase (*SARK*) gene was used to regulate the expression of the *IPT* gene. The reports revealed improved drought tolerance and increased seed yield in tobacco, rice and peanut under water-stressed conditions [1,11,12,23]. As a drought avoidance strategy, most plants close their stomata to minimize water loss, which also reduces CO_2_ uptake and hence decreases photosynthetic activity. Higher drought tolerance induced by increased cytokinin levels was suggested by enhanced photorespiration and prevention of degradation of photosynthetic protein complexes during periods of water stress [3,24]. Delayed leaf senescence in these plants due to cytokinin biosynthesis was regulated by the activation of *SARK* promoter in response to drought-induced leaf senescence. Drought stress is known to initiate leaf senescence and early maturation of most annual crop plants. Arguably, leaf senescence might be prematurely activated during drought, and the benefits of yield increase might not be achieved under well watered conditions using stress-inducible promoters. To overcome the issue of stress inductibility and appropriate regulation of the *IPT* gene, the present study used a developmental process-related promoter from the *AtMYB32* gene of *Arabidopsis thaliana*. Previously, the *AtMYB32* promoter was observed to direct the expression of a reporter gene to leaf and root vascular tissues, with some expression in the reproductive organs [25]. In this study, a modified version of the *AtMYB32* promoter was used (designated *AtMYB32xs*), in which the promoter motif that is specific for root expression was removed. The stable transgenic canola (*Brassica napus* L.) plants expressing *AtMYB32xs::IPT* exhibited a delay in leaf senescence under both controlled environment and field conditions. The transgenic plants produced a significantly higher seed yield under both rainfed and irrigated field conditions. Furthermore, no morphological or phenological abnormalities were observed in transgenic plants.

## Materials and Methods

### Transformation of canola plants

The transgenic canola plants were developed for the expression of a chimeric cytokinin biosynthesis gene; *Isopentenyl Transferase* (*IPT*) from *Agrobacterium tumefaciens* tmr-gene encoded by the octopine Ti plasmid under the control of a promoter from a developmentally regulated transcription factor gene *AtMYB32* (AT4G34990) from *Arabidopsis thaliana*. The transformation protocol used was as described previously by Lin et al. [25]. The *AtMYB32* gene is expressed ubiquitously in all parts of *Arabidopsis* plants, including the shoots, leaves, reproductive organs and roots (http://www.arabidopsis.org, and http://www.bar.utoronto.ca). It has previously been reported that the *AtMYB32* promoter directs the expression of the β-glucuronidase reporter gene to leaf and root vascular tissues and low expression in reproductive organs [25]. As higher cytokinin levels are required in the aerial portion of the plant as compared to roots, and to avoid any root formation abnormalities, the root-specific motif from the *AtMYB32* promoter was removed. The modified promoter was named as *AtMYB32xs* (S1 Fig.). The schematic diagram of *AtMYB32* promoter and modified *AtMYB32xs* promoter and sequences of promoter and *IPT* gene are presented in S1A, S1B, and S1C Fig.

The *AtMYB32xs::IPT* gene cassette was inserted into the cloning vector pPZP200 (S2 Fig.) and then transformed into *Agrobacterium* strain LBA4404. Hypocotyl explants of canola wild type (WT, i.e. non-transgenic) cv. RR014 were co-cultivated with *Agrobacterium* harbouring a binary vector for the expression of a chimeric *AtMYB32xs::IPT* gene as well as a chimeric *Hygromycin phosphotransferase* (*Hph*) gene as selectable marker. Hypocotyl explants were subjected to *in vitro* culture in presence of hygromycin to enable the recovery of putative transgenic plants. Homozygous transgenic canola plants were selected in T2 generation and were advanced to T4 generation for seed bulk-up for field evaluation. Southern hybridization analysis confirmed single copy insertion in the selected transgenic events (S3 Fig.).

### Molecular analysis of transgenic plants

Total RNA was isolated from plant tissue using Trizol (Gibco BRL). The cDNA was synthesized from total RNA by using the Reverse Transcription System kit (Promega). Real-time PCR was performed for the target gene *IPT*. Genomic DNA was extracted using a CTAB-based protocol. The transgenic plants were identified by production of a 375 bp PCR amplicon from the coding region of the *Hph* gene. PCR products were separated on 1% (w/v) agarose gels.

### Design of field experiments

The field experiments were conducted under the license DIR 103- limited and controlled release of canola genetically modified for enhanced yield and delayed leaf senescence, approved by the Office of the Gene Technology Regulator, Australia. Three transgenic lines; 6.6.38, 6.6.40 and 7.1.38 representing two independent events, two null control lines (plants which had passed through the transformation process but were confirmed as unmodified escapes); 6.2 and 7.6 and a WT line; cultivar RR014 were grown in field experiments. The field experiments were conducted for a single growing season at two separate geographic and environmental locations, Horsham (S 36°43’59” E 142°05’56”) and Hamilton (S 37°50’20” E 142°05’43”), these research farms are owned by the Department of Environment and Primary Industries, Victoria, Australia. These sites represent different soil and climatic conditions. Horsham has vertisol soils with cracking clay, medium rainfall with a temperate climate and Hamilton has chromosol soil with brown loamy topsoil, high rainfall and a temperate climate. The monthly rainfall for both experimental sites during the experimental period, along with long-term average rainfall patterns, is presented in Table A in S1 File.

The experimental design at Horsham was a split-plot design with four replicates each of rainfed and irrigated treatments as a main plot and genotypes as a sub-plot. Eight irrigation treatments (using a drip irrigation system) were applied during the reproductive growth phase, starting from onset of anthesis in the irrigated treatment plots. The timing and irrigation quantity is presented in Table B in S1 File. The experimental design at Hamilton was a complete randomised block design with six replicates. The distance between replicates was 2 m, each plot was 5 m in length, and the distance between plots within each replicate was 45 cm. The plot had six rows at 15 cm apart.

### Growth and yield observations

The following growth and yield observations were recorded:

#### Early season vigour

Early season vigour scores were determined on three occasions during the vegetative growth stage and were presented as the mean of all the observations. The score was based on a scale of 1 to 10, with 1 representing poor vigour, and 10 as excellent vigour. The plants were examined for overall growth, yellowing, stunting, crinkled or rolled leaves.

#### Phenology

The timings for budding, onset of flowering, cessation of flowering, and physiological maturity were recorded.

#### Senescence score

Plants in each plot were visually assessed for senescence (yellowing, browning and dying of leaves and colour change in silique from green to pale and light brown) at three time points during silique formation to physiological maturity. The scores were averaged, with 1 denoting green and 10 denoting complete senescence.

#### Days to maturity

Each plot was examined daily as physiological maturity approached. Days to maturity was determined by the change in silique colour, from green to yellow and light brown.

#### Seed yield

Seed harvested from each plot was weighed separately, yield being expressed as tons per hectare.

#### Seed quality

Seed quality analysis was performed on three replicate seed lots from each plot to determine percentage oil and protein content, glucosinolates (μmol/g) and fatty acid profiles (palmitic acid, stearic acid, oleic acid, linoleic acid, α-linolenic acid, arachidic acid, and eicosenoic acid) using a Near Infrared, XDS- Rapid Content Analyzer (FOSS NIRSystems, Inc.).

#### Silique shatter test

A custom designed pendulum machine was used to test silique shatter resistance, as described by Liu et al. [26]. This machine records rupture energy, which is directly proportional to silique shatter resistance. Ten to twelve mature silique from each plot were tested for silique shatter resistance.

#### SPAD Chlorophyll

Chlorophyll levels were measured at different time points post-flowering using a chlorophyll meter (SPAD 501, Konica Minolta). Chlorophyll levels were recorded on four positions per leaf, two leaves per plant and three plants per plot.

#### Normalised difference vegetation index (NDVI)

A Crop circle (ACS-210, Holland Scientific, Inc) was used to estimate NDVI by measuring reflectance at 680nm (visible region) and 900nm (near infra-red region). The NDVI index was then calculated using the formula (R_900_ − R_680_) / (R_900_+ R_680_), according to Gamon et al. [27]. Data was taken by scanning over the experimental plots at several time points from budding until physiological maturity.

### Statistics

All data were statistically analysed by analysis of variance (ANOVA) using GenStat statistical software (VSN International, Ltd).

## Results

### *AtMYB32xs::IPT* transgenic canola plants displayed delayed leaf senescence under controlled environment conditions

**Figure 1:**
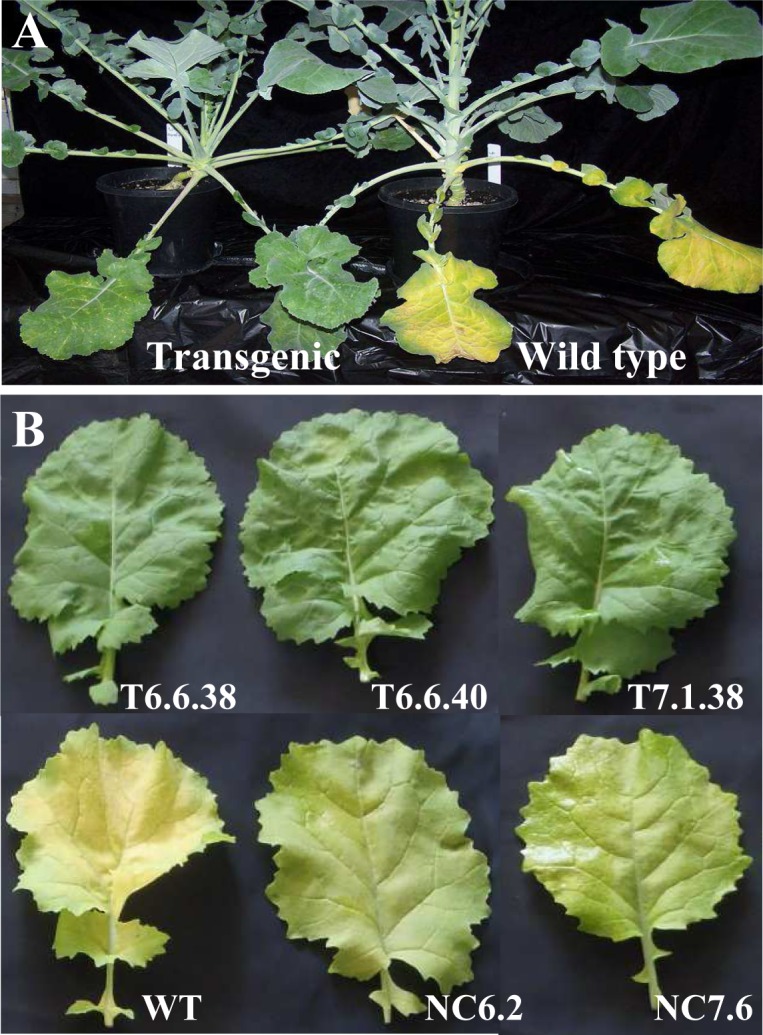
Leaf senescence in plants of canola genotypes grown under controlled environment conditions. **A**. Transgenic and wild type plants. **B**. Detached leaf assay on leaves of transgenic (6.6.38, 6.6.40 and 7.1.38), WT and null control (6.2 and 7.6) plants was done. Plants were grown for five weeks, leaves from similar position were picked and kept on moist filter paper for one week. NC, null control; T, transgenic, WT, wild type.

The PCR-positive transgenic plants for the *Hph* selective gene were also confirmed for the presence of *IPT* gene (S4A and S4B Fig.). The transgenic plants grown under controlled environment conditions demonstrated a delay in leaf senescence. At the reproductive growth stage, in which the lower leaves of WT plants started to senesce, leaves of transgenic plants remained green (Fig. 1A). To confirm this behaviour, leaves of similar age from transgenic, null control non-transgenic and WT plants were detached and placed on moist filter paper for a week. The transgenic leaves remained green during this period, while WT and null control leaves turned pale, an indication of senescence and chlorophyll breakdown (Fig. 1B). The seed yield in transgenic plants was higher than for WT and null control plants, grown under controlled environmental conditions (data not shown). These selected transgenic, null control and WT lines were further evaluated under field conditions, as described subsequently.

### Growth and phenology of canola genotypes under field conditions

The field experiments at Horsham and Hamilton, which represent medium and high rainfall zones, respectively. The total rainfall during the months of canola field experiments was lower than the long-term average rainfall at both sites. However, the Hamilton site received c. 1.6-times higher rainfall than the Horsham site (Table A in S1 File). The total rainfall for the month of September was substantially lower (<25%) than the long-term average at the Horsham site, which coincided with flowering and may have been a major factor for the significantly lower yield observed in rainfed plots as compared to irrigated plots. The first irrigation treatment was applied in September, with a total of eight treatments applied when lack of rainfall was deemed to be causing water stress (Table B in S1 File).

Early season vigour for the transgenic (6.6.38, 6.6.40, and 7.1.38) lines and null control (6.2, and 7.6) lines were within the range of 5.0 to 6.0 (Table 1 and Table C in S1 File), indicating comparable growth in both transformed and untransformed plants. Budding (initiation of inflorescence) occurred at 84–85 days after planting (DAP) in the transgenic lines 6.6.38 and 6.6.40 and the null control 6.2, while transgenic line 7.1.38 and null control 7.6 required 87 days (Table 2). Flowering began 3–4 days earlier at the Hamilton site (Table C in S1 File) than at the Horsham site (Table 2). Between genotypes, the transgenic plants flowered 1–2 days earlier than the nulls at the Hamilton site (Table C in S1 File), although there was no difference in flowering times for genotypes located at the Horsham site (Table 2). Flowering persisted eight days longer at the Hamilton site than at the Horsham site, due to relatively moister conditions at Hamilton. There was little difference between the transgenic plants and null controls for flowering duration within sites (Table 2 and Table B in S1 File). The timely availability of water in irrigated treatments at Horsham was reflected in effects on phenology: plants in rainfed plots completed flowering c. 2 days earlier than the irrigated plants (Table 2). Physiological maturity in the transgenic plants was delayed by two days compared to the null control lines at both sites (Table 2 and Table C in S1 File). The difference in physiological maturity between the rainfed and irrigated plants was c. 2 days, such that rainfed plants matured earlier (Table 2). Early life-cycle completion is a known phenomenon in water-stressed plants.

**Table 1:** Early season vigour and senescence score in canola genotypes at the Horsham field experiment.

**Genotype**	**Early season vigour**	**Senescence score**	
		**Irrigated**	**Rainfed**
**T6.6.38**	5.5	3.5*	4.4
**T6.6.40**	5.0	3.6*	4.3
**T7.1.38**	6.0	3.1*	4.0
**NC6.2**	5.5	4.9	5.9
**NC7.6**	6.0	3.8	4.6
**WT**	4.5	4.8	5.8

NC, Null control; T, Transgenic; WT, wild type.

* Values significantly different in transgenic line than corresponding null at P < 0.05.

**Table 2:** Phenology in canola genotypes at the Horsham field experiment.

**Genotype**	**Budding (DAP)**	**Flowering start (DAP)**	**Flowering end (DAP)**		**Physiological maturity (DAP)**	
			**Irrigated**	**Rainfed**	**Irrigated**	**Rainfed**
**T6.6.38**	85	111	141	139	190	188
**T6.6.40**	85	111	141	139	190	188
**T7.1.38**	87	111	141	140	190	188
**NC6.2**	84	112	141	139	188	186
**NC7.6**	87	111	142	140	189	187
**WT**	84	112	142	141	189	187

DAP, days after planting, NC, Null control; T, Transgenic; WT, wild type

### Transgenic lines displayed delayed leaf senescence and enhanced green canopy cover under field conditions

**Figure 2:**
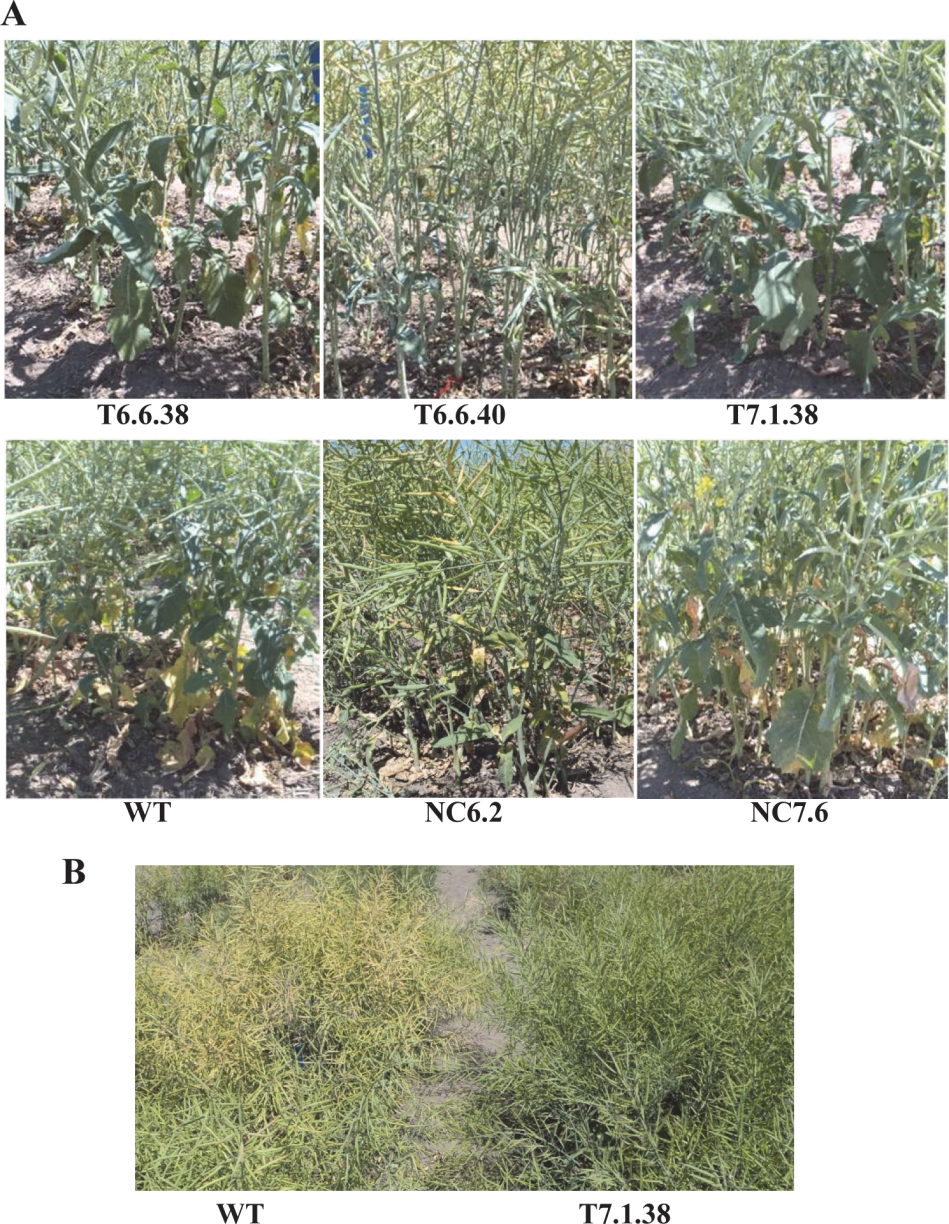
Senescence progression in canola genotypes near maturity under field conditions. **A**. Basal plant parts. **B**. Crop canopy in experimental plots. Pictures were taken at 174 DAP. DAP, days after planting; NC, null control; T, transgenic, WT, wild type.

The aim of the field evaluation experiments was to assess delayed leaf senescence trait in transgenic lines mediated by the expression of the cytokinin biosynthesis gene; *IPT* and whether this trait was correlated with increased seed yield. During the period from silique setting to physiological maturity, plants were regularly observed for leaf senescence and silique colour change from green to pale and light brown. All the transgenic lines (6.6.38, 6.6.40, and 7.1.38) obtained low scores when compared to null control lines 6.2 and the WT control at both the Horsham and Hamilton sites (Table 1 and Table C in S1 File). As expected, plants in irrigated plots were greener, with lower leaf senescence scores than for rainfed plants (Table 1). Among the genotypes, transgenic plants exhibited a larger green leaf area, while non-transgenic and WT control plants displayed a greater proportion of leaves that turned pale (Fig. 2A). A comparison of plant canopies also showed that 7.1.38 transgenic plants were delayed in senescence, such that most silique were still greener when compared to WT plants (Fig. 2B).

**Figure 3:**
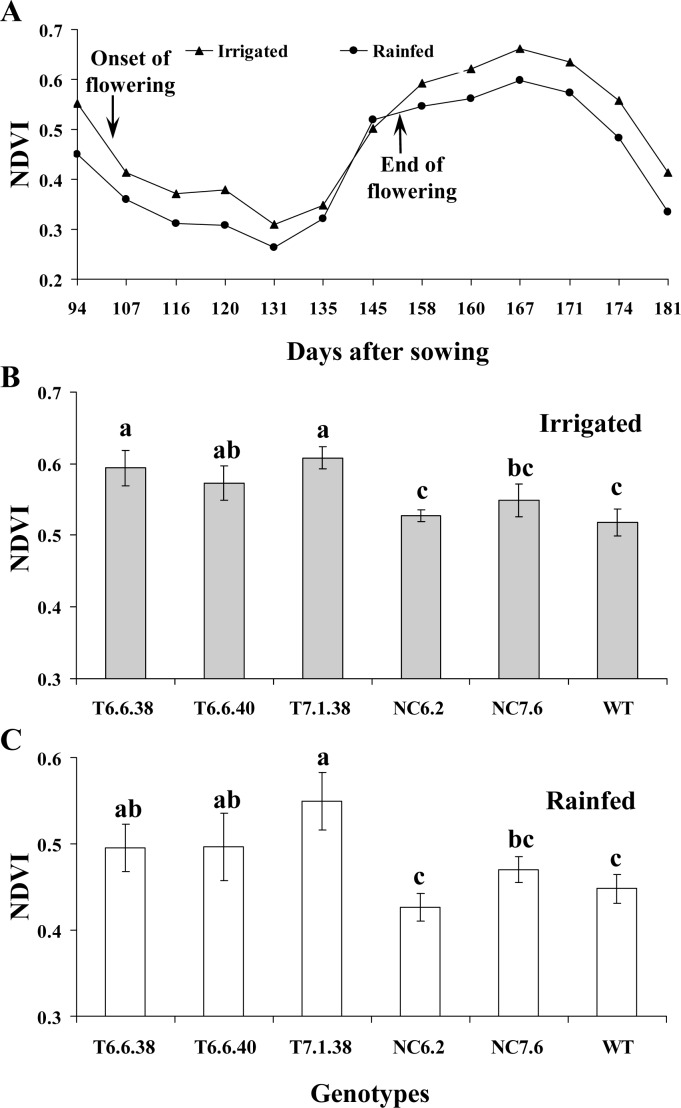
NDVI in canola genotypes under irrigated and rainfed field conditions. **A**. NDVI was calculated from data taken at different time points during the reproductive growth using crop circle. **B,C**. NDVI for transgenic (6.6.38, 6.6.40 and 7.1.38), null control (6.2 and 7.6) and WT at 174 days after sowing under irrigated and rainfed treatments. Data is mean ± SD. Bars with different letters indicate significant difference at P<0.05. DAP, days after planting; NDVI, Normalised difference vegetation index, NC, null control; T, transgenic, WT, wild type.

The NDVI data, which represents biomass/canopy cover and chlorophyll reflectance, was obtained using a crop circle equipment. The NDVI values exhibited a trough during flowering because of the propensity of yellow flowers to obscure the photosynthetic region of the canopy from the sensing unit, but thereafter NDVI values increased following completion of flowering (until 167 DAP), decreasing again due to onset of senescence and loss of photosynthates (Fig. 3A). Irrigated plants displayed higher NDVI values than rainfed plants during the period of data collection, with the exception at completion of flowering, as rainfed plants exposed their photosynthetic canopy earlier (Fig. 3A). Transgenic plants had higher NDVI values compared to null controls and the WT line (Fig. 3B, and 3C).

**Figure 4:**
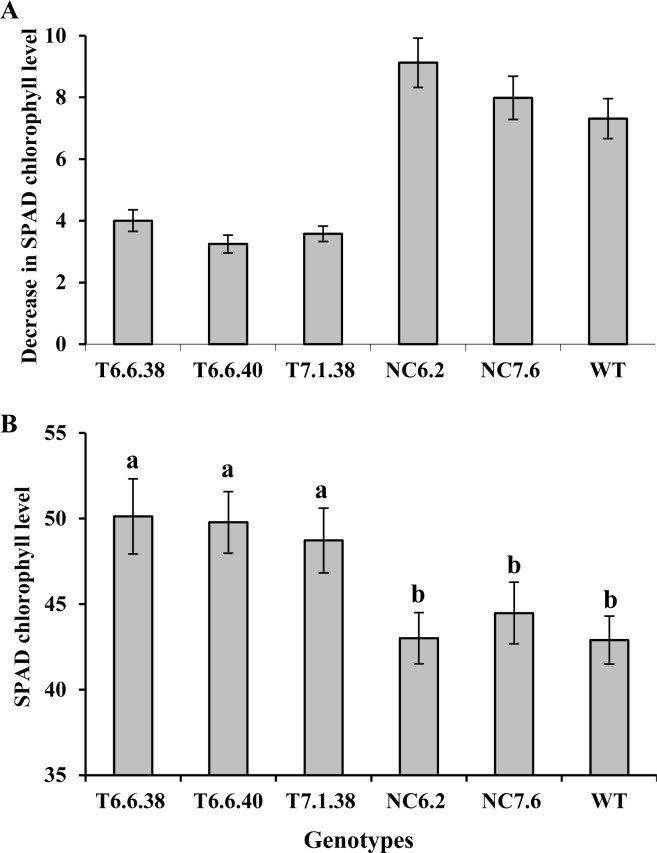
SPAD chlorophyll levels in canola genotypes under field conditions. **A**. Decrease in chlorophyll level in transgenic (6.6.38, 6.6.40 and 7.1.38), null control (6.2 and 7.6) and WT during ripening period; onset of flowering (150 DAP) till near to maturity (178 DAP). **B**. Chlorophyll level in genotypes at 178 days after sowing. Data is mean ± SD. Bars with different letters indicate significant difference at P<0.05. NC, null control; T, transgenic, WT, wild type.

Chlorophyll levels were recorded in leaves at multiple time points. Differences of chlorophyll content at completion of flowering (150 DAP) and at the onset of physiological maturity (178 DAP) is presented (Fig. 4A). The decrease in chlorophyll level was lower during the reproductive growth period in transgenic plants compared to null and WT controls (Fig. 4A). Additionally, the SPAD chlorophyll level in all transgenic lines was higher than for nulls and WT (Fig. 4B). The NDVI and SPAD data indicate that transgenic plants maintained higher green canopy cover and elevated chlorophyll levels for a longer duration compared to the null and WT control plants.

### Transgenic plants showed enhanced seed yield

The transgenic lines showed significantly higher seed yield compared to null control and WT plants under both irrigated and rainfed treatments at the Horsham field experiment (Table 3). The transgenic plants had higher number of flowers and siliques compared to null control and WT (data not shown). The seed yield differences between transgenic lines and the null control lines were more pronounced in rainfed environments (Table 3). Similarly, the transgenic lines obtained higher seed yield in comparison to null control and WT plants at the Hamilton site (Table 4). Among the transgenic lines, 7.1.38 performed best for seed yield, followed by lines 6.6.38 and 6.6.40 (Table 3, 4). The percentage increases in seed yield in transgenic plants over their corresponding null control lines at the Horsham field experiment were determined. For irrigated treatments, transgenic lines 6.6.38 and 6.6.40 exceeded the corresponding null control line 6.2 by 8.3% and 6.9%, respectively, while transgenic line 7.1.38 showed 15.9% greater yield than the corresponding null control line 7.6 (Table 3). For rainfed treatment, transgenic lines 6.6.38 and 6.6.40 exceeded the corresponding null control line 6.2 by 23.2% and 15.9%, respectively, while transgenic line 7.1.38 showed 21.1% greater yield than the corresponding null control line 7.6 in the rainfed treatment (Table 3). The plants at the Hamilton site showed an increase of 18.7% (transgenic line 6.6.38) and 13.7% (transgenic line 6.6.40) compared to their corresponding null control line 6.2. An increase of 14.7% in transgenic line 7.1.38 was also observed compared to the corresponding null control line 7.6 (Table 4).

**Table 3:** Seed yield in canola genotypes at the Horsham field experiment.

**Genotype**	**Seed Yield (t/ha)**			**% yield increase over corresponding null**	
	**Irrigated**	**Rainfed**	**Average**	**Irrigated**	**Rainfed**
**T6.6.38**	3.99*	3.10*	3.55	8.3	23.2
**T6.6.40**	3.93*	2.83*	3.38	6.9	15.9
**T7.1.38**	4.29*	3.23*	3.76	15.9	21.1
**NC6.2**	3.66	2.38	3.02		
**NC7.6**	3.61	2.55	3.08		
**WT**	3.39	2.28	2.84		
					
Statistics	Irrigation	Genotype	Irrg x Gen		
SED	0.092	0.161	0.259		
LSD (5%)	0.225	0.332	0.519		
P Value	<.001	<.001	0.952		

SED; standard error of difference of means; LSD; least significant difference; NC, Null control; T, Transgenic; WT, wild type

* Yield significantly different in transgenic line than corresponding null.

Yield increase (%) of transgenic lines 6.6.38 and 6.6.40 over corresponding null control 6.2 is shown. Yield increase (%) of transgenic line 7.1.38 over corresponding null control 7.6 is shown.

**Table 4:** Seed yield at the Hamilton field experiment.

**Genotype**	**Seed Yield (t/ha)**	**% yield increase over corresponding null**
**T6.6.38**	5.04*	18.7
**T6.6.40**	4.75*	13.7
**T7.1.38**	5.18*	14.7
**NC6.2**	4.10	
**NC7.6**	4.42	
**WT**	3.82	

Yield Statistics: Standard error of difference of means (SED)- 0.366, LSD (5%); least significant difference- 0.735, P value <0.001.

* Yield significantly different in transgenic line than corresponding null.

* Yield significantly different in transgenic line than corresponding null.

Yield increase (%) of transgenic lines 6.6.38 and 6.6.40 over corresponding null control 6.2 is shown. Yield increase (%) of transgenic line 7.1.38 over corresponding null control 7.6 is shown.

It was observed that during the ripening period, silique shattering in non-transgenic plants was higher than for transgenic plants. For this evaluation, silique were tested in a custom-designed machine which records rupture energy, a property directly proportional to silique shatter resistance. Results (Table 5) indicate that silique of all transgenic lines exhibited significantly lower rupture energy than for null control and WT lines.

**Table 5:** Silique shatter resistance measured as rupture energy (mJ) in canola genotypes at the Horsham field experiment.

**Genotype**	**Rupture Energy (mJ)**		
	**Irrigated**	**Rainfed**	**Average**
**T6.6.38**	3.00*	2.89*	2.94
**T6.6.40**	3.02*	2.87*	2.95
**T7.1.38**	2.84*	2.68*	2.76
**NC6.2**	2.80	2.65	2.72
**NC7.6**	2.60	2.50	2.55
**WT**	2.54	2.45	2.50
			
Statistics	Irrigation	Genotype	Irr x Gen
SED	0.062	0.113	0.202
LSD (5%)	0.151	0.237	0.404
P Value	0.118	<.001	1.00

SED, standard error of difference of means; LSD; least significant difference; NC, Null control; T, Transgenic; WT, wild type.

* Rupture energy significantly different in transgenic line than corresponding null.

### Transgenic lines exhibited improved and/or sustained seed quality parameters

A range of seed quality parameters were analysed, including oil, protein, and glucosinolate contents as well as fatty acid profiles (Tables 6–8 and Table D in S1 File). Oil content was highest in transgenic line 7.1.38 compared to nulls and WT for both irrigated and rainfed treatments at the Horsham site (Table 6). A similar pattern was observed for oil content at the Hamilton site, although the differences observed between genotypes were not statistically significant (Table D in S1 File). Oil content was higher in plants from the irrigated treatment to those from the rainfed treatment within the Horsham-based experiment (Table 6).

**Table 6:** Seed quality parameters; moisture, oil, glucosinolate and protein contents in canola genotypes at the Horsham field experiment.

**Genotype**	**Oil Content (%)**			**Glucosinolate (μmol/g)**			**Protein Content (%)**		
	**Irrigated**	**Rainfed**	**Average**	**Irrigated**	**Rainfed**	**Average**	**Irrigated**	**Rainfed**	**Average**
**T6.6.38**	45.0	45.3	45.1	11.7	12.7	12.2	22.6	22.9	22.8
**T6.6.40**	44.8	44.6	44.7	12.2	14.9	13.6	22.3	23.1	22.7
**T7.1.38**	46.5*	45.6*	46.1	6.6	7.1	6.9	20.8*	22.6*	21.7
**NC6.2**	45.6	44.9	45.2	11.0	13.8	12.4	22.2	22.7	22.5
**NC7.6**	45.3	44.1	44.7	7.2	7.8	7.5	22.0	23.5	22.8
**WT**	46.4	44.6	45.5	8.0	8.8	8.4	21.1	23.2	22.2
									
Statistics	Irrigation	Genotype	Irrg x Gen	Irrigation	Genotype	Irrg x Gen	Irrigation	Genotype	Irrg x Gen
SED	0.376	0.352	0.436	0.196	0.343	0.500	0.178	0.277	0.412
LSD (5%)	0.975	0.905	0.890	0.480	0.688	1.001	0.435	0.555	0.825
P Value	0.013	<.001	0.002	0.001	<.001	0.01	<.001	<.001	0.040

SED, standard error of difference of means; LSD, least significant difference; NC, Null control; T, Transgenic; WT, wild type.

* Values significantly different in transgenic line than corresponding null.

**Table 7:** Seed quality parameters; fatty acids (% of oil content) in canola genotypes at the Horsham field experiment.

**Genotype**	**Palmitic Acid (16:0)**			**Stearic Acid (18:0)**			**Oleic Acid (18:1)**			**Linoleic Acid (18:2)**		
	**Irrigated**	**Rainfed**	**Average**	**Irrigated**	**Rainfed**	**Average**	**Irrigated**	**Rainfed**	**Average**	**Irrigated**	**Rainfed**	**Average**
**T6.6.38**	3.88	3.88	3.88	3.48	3.42	3.45	62.4	63.2	62.8	14.1	13.8	13.9
**T6.6.40**	3.93	3.92	3.93	3.43	3.53	3.48	62.0	62.0	62.0	14.6	14.6	14.6
**T7.1.38**	3.93	3.87	3.90	3.55	3.35	3.45	62.7*	62.4*	62.6	13.8*	14.3*	14.0
**NC6.2**	3.86	3.82	3.84	3.41	3.43	3.42	61.9	62.1	62.0	14.0	14.1	14.0
**NC7.6**	3.85	3.88	3.87	3.46	3.41	3.43	61.4	61.0	61.2	14.7	15.2	14.9
**WT**	3.83	3.84	3.84	3.81	3.70	3.76	64.1	62.8	63.4	11.7	13.2	12.5
												
Statistics	Irrigation	Genotype	Irrg x Gen	Irrigation	Genotype	Irrg x Gen	Irrigation	Genotype	Irrg x Gen	Irrigation	Genotype	Irrg x Gen
SED	0.015	0.036	0.026	0.040	0.059	0.088	0.290	0.300	0.500	0.250	0.320	0.500
LSD (5%)	0.038	0.092	0.054	0.097	0.118	0.177	0.710	0.610	1.010	0.620	0.640	1.000
P Value	0.955	<.001	0.117	0.176	<.001	0.36	0.307	<.001	0.0	0.101	<.001	0.3

SED, standard error of difference of means; LSD, least significant difference; NC, Null control; T, Transgenic; WT, wild type.

* Values significantly different in transgenic line than corresponding null.

**Table 8:** Seed quality parameters; fatty acids (% of oil content) in canola genotypes at the Horsham field experiment.

**Genotype**	α-**Linolenic Acid (18:3)**			**Arachidic Acid (20:0)**			**Eicosenoic Acid (20:1)**		
	**Irrigated**	**Rainfed**	**Average**	**Irrigated**	**Rainfed**	**Average**	**Irrigated**	**Rainfed**	**Average**
**T6.6.38**	13.7	13.5	13.6	0.93	0.91	0.92	1.13	1.13	1.13
**T6.6.40**	13.7	13.5	13.6	0.91	0.92	0.92	1.12	1.14	1.13
**T7.1.38**	13.7	13.5	13.6	0.92	0.90	0.91	1.10	1.12	1.10
**NC6.2**	14.1	14.0	14.0	0.91	0.93	0.92	1.12	1.14	1.13
**NC7.6**	13.8	13.8	13.8	0.94	0.93	0.93	1.13	1.16	1.14
**WT**	13.8	13.9	13.8	1.02	1.01	1.01	1.10	1.14	1.12
									
Statistics	Irrigation	Genotype	Irrg x Gen	Irrigation	Genotype	Irrg x Gen	Irrigation	Genotype	Irrg x Gen
SED	0.100	0.190	0.210	0.006	0.017	0.023	0.009	0.01	0.010
LSD (5%)	0.250	0.480	0.430	0.015	0.033	0.046	0.012	0.03	0.020
P Value	0.623	<.001	0.934	0.882	<.001	0.838	0.001	<.001	0.06

SED, standard error of difference of means; LSD, least significant difference; NC, Null control; T, Transgenic; WT, wild type

Glucosinolates are known for their toxic effects to human and animal in high doses. The use of glucosinolate-containing crops as a primary food source for animals has also been shown to have negative effects on livestock health. Canola varieties with low glucosinolate content would be preferable from a health perspective. Glucosinolate contents were lowest in the transgenic line 7.1.38 in both the Horsham and Hamilton experiments (Table 6 and Table D in S1 File). Oil and protein contents are generally inversely proportional to one other. The protein content in the transgenic line 7.1.38 was lower compared to the nulls, but statistically similar to WT in the Horsham experiment (Table 6), but no differences were observed between transgenic lines and the null controls in the Hamilton experiment (Table D in S1 File). The protein content was lower for irrigated than for rainfed plants (Table 6).

Palmitic and stearic acids are saturated fatty acids. There was little difference between the transgenic lines and the null control lines for these fatty acids (Table 7 and Table D in S1 File). Oleic acid (omega 9), an unsaturated fatty acid, was higher in the transgenic lines compared to the null control lines (Table 7 and Table D in S1 File). Linoleic acid (omega 6), a poly-unsaturated fatty acid was lower in the transgenic line 7.1.38 compared to the null control 7.6 (Table 7 and Table D in S1 File). The α-linolenic acid (omega 3), a poly-unsaturated fatty acid, displayed no contrasting difference among transgenic and null control lines (Table 8 and Table D in S1 File). The arachidic (saturated) and eicosenoic (unsaturated) fatty acids were at low levels compared to the other fatty acids. There was little difference in arachidic acid level between transgenic and null control lines (Table 8 and Table D in S1 File). Eicosenoic acid was slightly lower in the transgenic line 7.1.38 compared to the null control 7.6 (Table 8 and Table D in S1 File).

## Discussion

Among abiotic stresses, water stress is of primary concern and is a major limiting factor for crop production on a global basis. Episodes of drought are expected to be more frequent and severe in the future. In addition, increasing agricultural output will be under pressure to meet the food demands of a rising human population [28,29]. In the present study, the objective was to develop transgenic plants with higher yield under conditions of limited water availability as well as superior performance under normal water conditions. Expression of the *IPT* gene driven by a modified promoter *AtMYB32xs* delayed leaf senescence in transgenic plants, resulting in higher seed yield than for the null controls and WT plants under both controlled environment and field grown conditions. Water stress at the reproductive growth stage has particularly severe consequences for yield, and is prevalent in crops grown under most water stressed conditions [30]. In the field experiment located at Horsham, water availability to plants undergoing rainfed treatment during reproductive growth was low, due to minimal rainfall during this period. This was the major factor resulting in significant yield reduction in rainfed plants as compared to those experiencing irrigated treatments (Table 3). However, the yield reduction in transgenic plants experiencing identical water stress conditions was much lower. Interestingly, the transgenic plants produced greater seed yield under both rainfed and irrigated field conditions than null control and WT plants (Table 3). Nonetheless, yield increase in transgenic plants was intensified, with a larger increase under rainfed conditions (up to 23%), but also with a significant yield increase (up to 16%) under irrigated conditions. Transgenic tobacco, peanut and rice plants developed using the *IPT* gene under the control of the *SARK* promoter have shown seed yield increases only under water-stressed conditions, but no difference in yield in well-watered control treatments [1,11,12]. Notably, the *SARK* promoter is activated by initiation of drought-induced leaf senescence, and so yield benefits were observed only under drought conditions. The *IPT* gene, driven by a senescence inducible promoter *SAG12* was introduced into crop plants such as wheat leading to increased nitrate flux and nitrate reductase activity [2], and cassava with increased drought tolerance [20]. A similar senescence-enhanced promoter, *SEE1*, when controlling the *IPT* gene in maize exhibited the ‘stay-green’ trait [10]. These studies reported a delay in leaf senescence, but no yield benefits were observed. This effect was attributed to the assumption that the delay in leaf senescence also served to delay translocation of metabolites from leaves to reproductive organs, which in turn failed to contribute to yield increases in wheat plants [2]. In addition, feedback inhibition of cytokinin due to activation of *SAG12* promoter by the onset of senescence, but repression upon delaying leaf senescence, may have occurred. Clearly, selection of a promoter which can regulate optimal *IPT* gene expression is critical for obtaining economic benefits from transgenic plants. This is the first report in which selection and modification of a promoter (*AtMYB32xs*) to drive the *IPT* gene has obtained beneficial effects on yield regardless of water availability. The delayed leaf senescence in transgenic canola plants correlates well with higher NDVI in their respective field plots (Fig. 3) and maintenance of chlorophyll levels for an extended period (Fig. 4).

Furthermore, no penalty on growth or phenology was observed in transgenic plants. Budding and flowering occurred simultaneously in all genotypes that were evaluated under field conditions. Growth duration was also similar, with a minor delay of 1–2 days for physiological maturity in transgenic plants (Table 2). Silique shattering is a major concern in canola during maturity, and can cause up to 50% seed loss [31]. Transgenic plants exhibited lower levels of silique shattering than null controls and WT plants (Table 5). Delayed leaf senescence may be a contributor in maintenance of silique strength in transgenic plants. Enhancement of cytokinin biosynthesis in *AtMYB32xs::IPT* transgenic plants could also be a possible factor for reduction of silique shattering. Adenosine Kinase (ADK) contributes to inter-conversion of cytokinin and impairment of silique shattering in *ADK* mutant *Arabidopsis* plants [32]. Consequently, it would be of great potential benefit to further explore the molecular and physiological roles of cytokinin in silique shattering of Brassicaceae plants.

As an oilseed crop, seed quality parameters are important in canola. Most of the quality parameters in transgenic seeds were maintained at similar levels to null control and WT seeds. Notably, oleic acid was higher in all transgenic lines, and in one of the transgenic lines higher oil content and lower glucosinolate contents were observed (Tables 6 to 8). Higher seed yield and improved seed quality in transgenic plants could be attributed to efficient and increased translocation of source metabolites towards developing seeds through maintenance of green photosynthetic source tissues for a longer duration. Understanding the molecular basis of this improvement due to delayed senescence, and the underlying role of cytokinin in the process, would be a beneficial research direction.

In conclusion, we have demonstrated that expression of *AtMYB32xs::IPT* has delayed the leaf senescence and improved seed yield under both low water availability (rainfed) and irrigated field conditions in an important oilseed crop, canola. A similar approach can be applied to other crops such as cereals, pulses, fibre and forages in order to harness the potential of increased productivity worldwide. It is encouraging that the transgenic plants showed significantly improved seed yield under rainfed conditions, contributing to efficient use of irrigation water, and thus ensuring farmers obtain superior yields. The same gene construct could produce beneficial results under good rainfall or irrigated conditions, without restricting usage to conditions of low water availability only.

## Supporting Information

S1 Fig Modification of the *AtMYB32* promoter. (TIF)

S2 Fig Gene cassette used to develop canola transgenic lines. (TIF)

S3 Fig Southern hybridisation analysis of transgenic T2 canola plants. (TIF)

S4 Fig Transgene confirmation in transgenic plants. (TIF)

S1 File Tables A-D. **Table A**. Rainfall (mm) pattern during experiments and long term rainfall average. **Table B**. Timing and amounts of irrigation applied at the Horsham field experiment. **Table C**. Early season vigour, senescence score and occurrence of reproductive growth stages in canola genotypes at the Hamilton field experiment. **Table D**. Seed quality parameters; moisture, oil, glucosinolate, protein and fatty acids contents in canola genotypes at the Hamilton field experiment.
(DOCX)
