# Supplementary figures and images for: Regulated Expression of a Cytokinin Biosynthesis Gene IPT Delays Leaf Senescence and Improves Yield under Rainfed and Irrigated Conditions in Canola (Brassica napus L.)
Source: PLoS One. 2015 Jan 20;10(1):e0116349. doi: 10.1371/journal.pone.0116349 (PMC4300212; doi:10.1371/journal.pone.0116349)

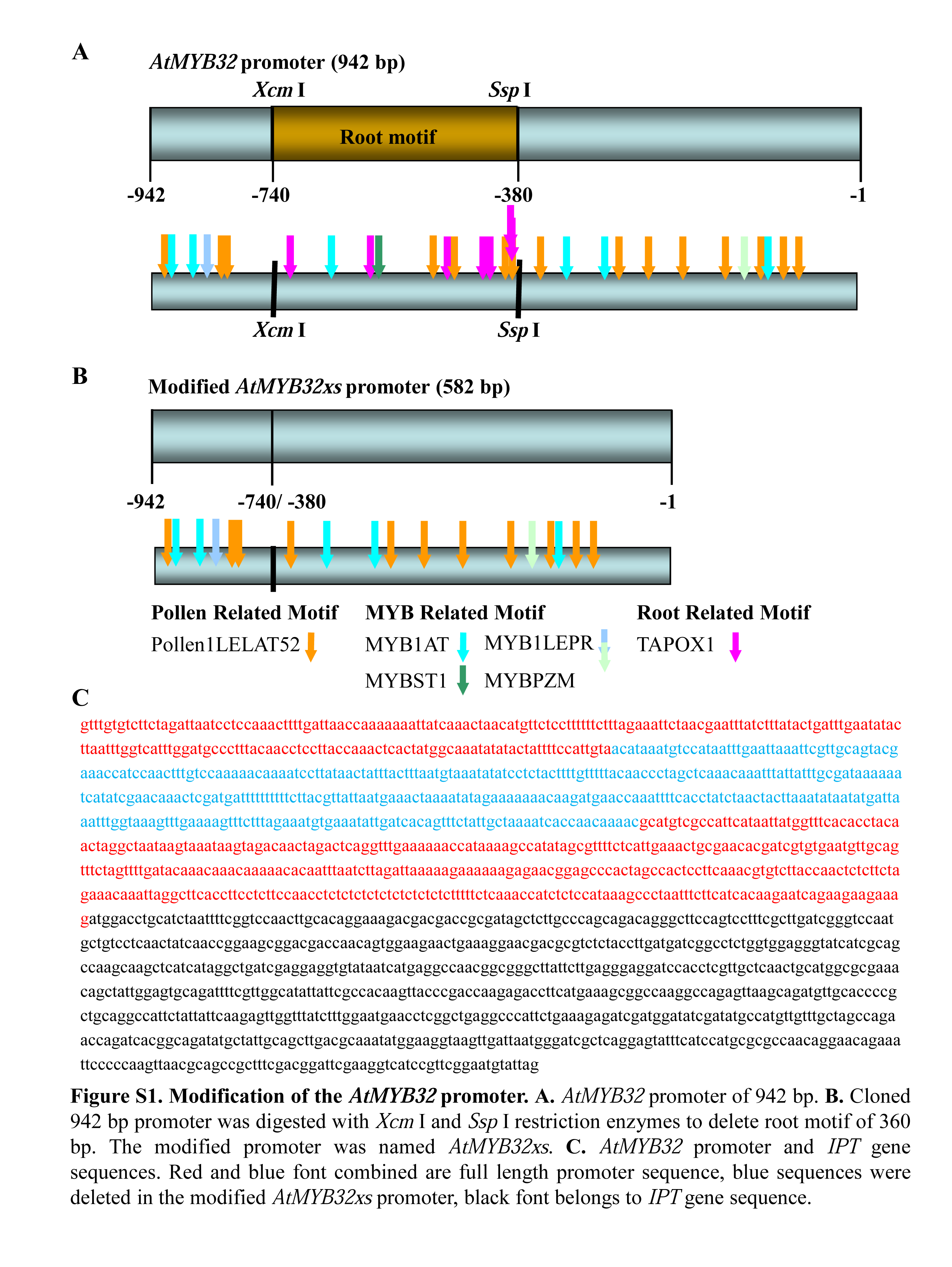

Supplement: S1 Fig — (TIF) [file pone.0116349.s001.tif]

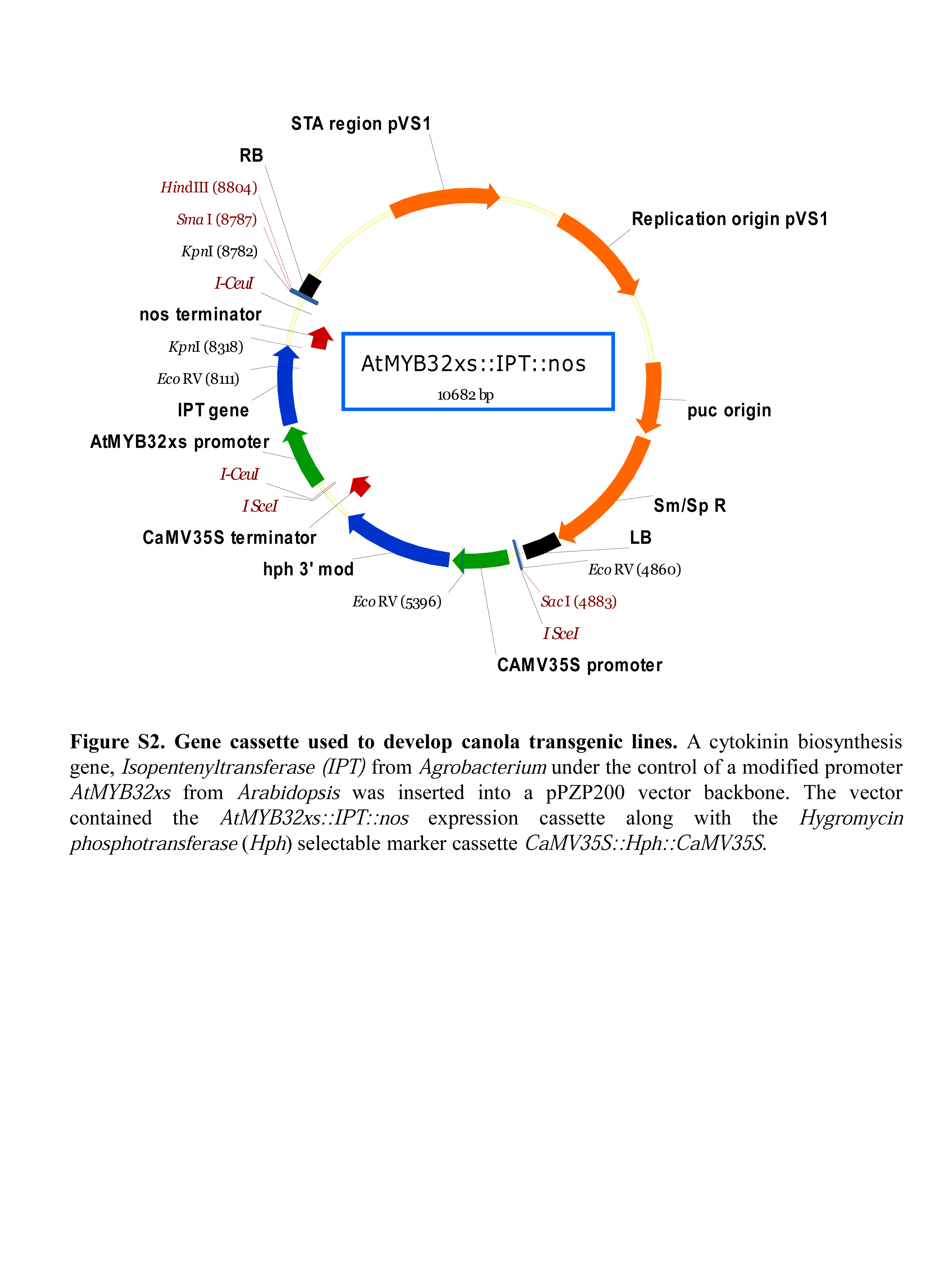

Supplement: S2 Fig — (TIF) [file pone.0116349.s002.tif]

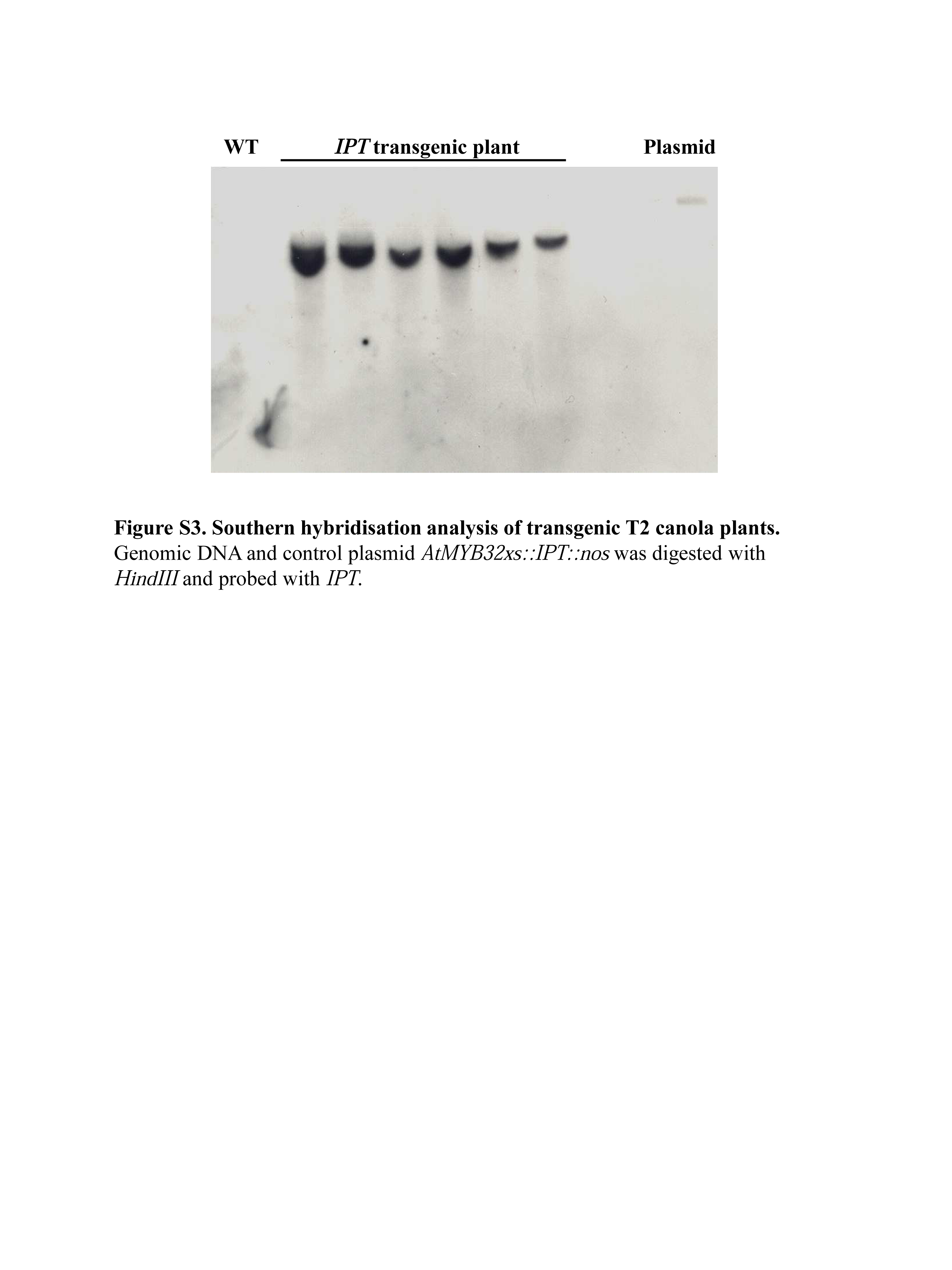

Supplement: S3 Fig — (TIF) [file pone.0116349.s003.tif]

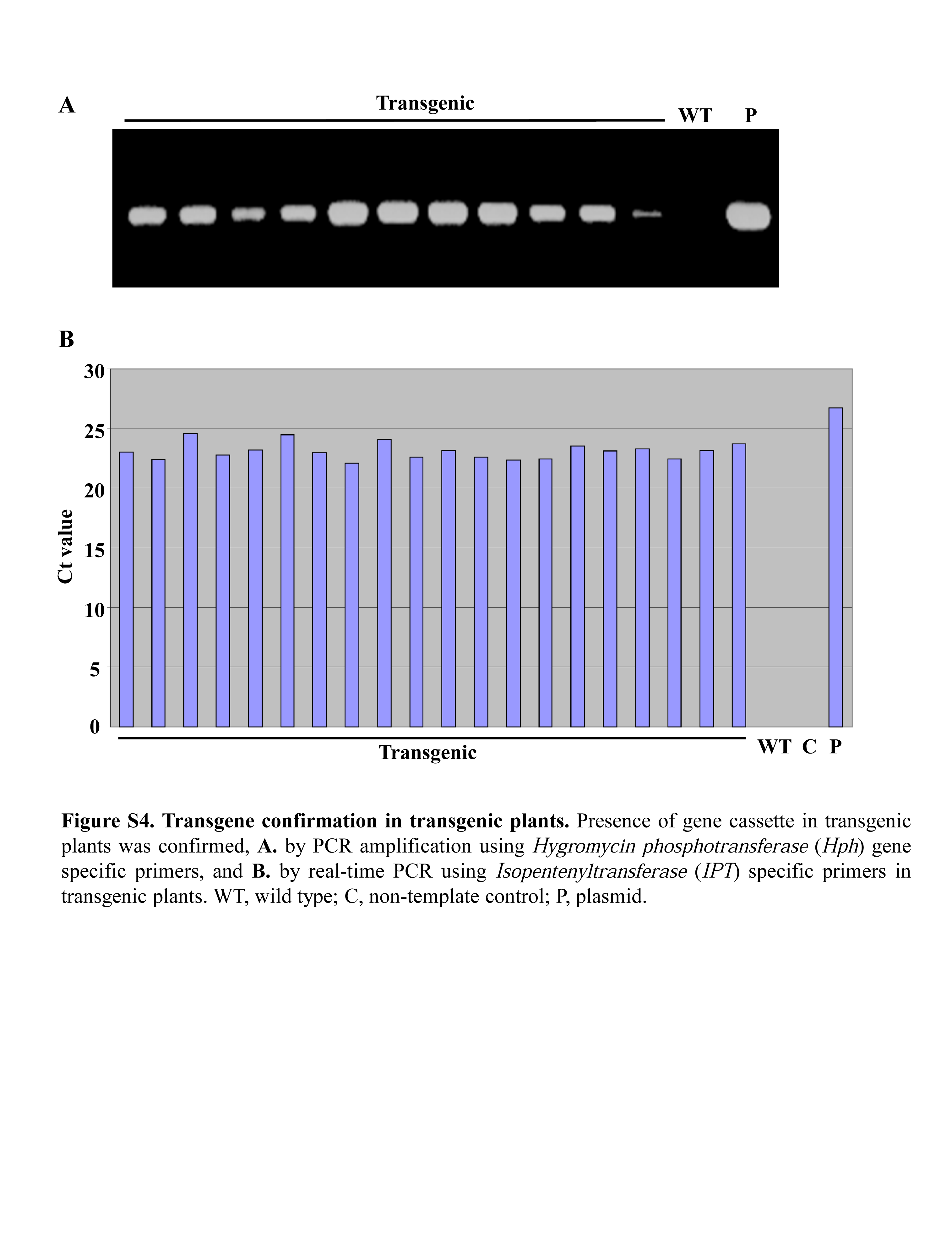

Supplement: S4 Fig — (TIF) [file pone.0116349.s004.tif]
