# Supplementary material for: Regulated Expression of a Cytokinin Biosynthesis Gene IPT Delays Leaf Senescence and Improves Yield under Rainfed and Irrigated Conditions in Canola (Brassica napus L.)
Source: PLoS One. 2015 Jan 20;10(1):e0116349. doi: 10.1371/journal.pone.0116349 (PMC4300212; doi:10.1371/journal.pone.0116349)
Supplement: S1 File — Table A. Rainfall (mm) pattern during experiments and long term rainfall average. Table B. Timing and amounts of irrigation applied at the Horsham field experiment. Table C. Early season vigour, senescence score and occurrence of reproductive growth stages in canola genotypes at the Hamilton field experiment. Table D. Seed quality parameters; moisture, oil, glucosinolate, protein and fatty acids contents in canola genotypes at the Hamilton field experiment. (DOCX) [file pone.0116349.s005.docx]

**Table A. Rainfall (mm) pattern during experiments and long term rainfall average.**

|  | **May** | **Jun** | **Jul** | **Aug** | **Sept** | **Oct** | **Till harvest (1-24 Nov)** | **Total** |
| --- | --- | --- | --- | --- | --- | --- | --- | --- |
| **Horsham** | | | | | | | | |
| **Experimental site** | 22.2 | 16.8 | 54.8 | 44.8 | 10.4 | 33.8 | 22.6 | 237.6 |
| **Long term average** | 43.0 | 38.2 | 47.1 | 47.9 | 44.0 | 42.2 | 33.5 | 295.9 |
| **Hamilton** | | | | | | | | |
| **Experimental site** | 45.8 | 59.5 | 70.0 | 61.5 | 39.5 | 62.1 | 44.2 | 382.6 |
| **Long term average** | 48.4 | 65.1 | 67.5 | 78.7 | 68.9 | 57.2 | 49.2 | 435.0 |

**Table B. Timing and amounts of irrigation applied at the Horsham field experiment.**

| **DAP** | **Irrigation (mm)** |
| --- | --- |
| 118 | 20 |
| 123 | 32 |
| 139 | 30 |
| 143 | 25 |
| 146 | 30 |
| 165 | 25 |
| 167 | 30 |
| 178 | 25 |
| Total | 217 |

DAP, days after planting.

**Table C. Early season vigour, senescence score and occurrence of reproductive growth stages in canola genotypes at the Hamilton field experiment.**

| **Genotype** | **Early season vigour** | **Senescence score** | **Flowering start (DAP)** | **Flowering end (DAP)** | **Physiological maturity (DAP)** |
| --- | --- | --- | --- | --- | --- |
| **T6.6.38** | 5.5 | 3.9* | 108 | 148 | 190 |
| **T6.6.40** | 5.5 | 3.8* | 109 | 149 | 190 |
| **T7.1.38** | 6.0 | 3.3* | 108 | 150 | 190 |
| **NC6.2** | 5.0 | 5.3 | 110 | 149 | 188 |
| **NC7.6** | 5.0 | 4.2 | 109 | 150 | 189 |
| **WT** | 5.0 | 5.2 | 110 | 150 | 189 |

DAP, days after planting, NC, Null control; T, Transgenic; WT, wild type. * Values significantly different in transgenic line than corresponding null at P < 0.05.

**Table D. Seed quality parameters; moisture, oil, glucosinolate, protein and fatty acids contents in canola genotypes at the Hamilton field experiment.**

| **Genotype** | **Oil (%)** | **Glucosin-olate (µmol/g)** | **Protein (%)** | **Palmitic Acid (16:0)** | **Stearic Acid (18:0)** | **Oleic Acid (18:1)** | **Linoleic Acid (18:2)** | α**-Linolenic Acid (18:3)** | **Arachidic Acid (20:0)** | **Eicosenoic Acid (20:1)** |
| --- | --- | --- | --- | --- | --- | --- | --- | --- | --- | --- |
| **T6.6.38** | 47.1 | 9.0 | 21.3 | 3.79 | 3.65 | 62.4 | 13.5 | 14.3 | 0.91 | 1.07 |
| **T6.6.40** | 46.4 | 10.6 | 21.3 | 3.84 | 3.66 | 61.6 | 14.1 | 14.2 | 0.92 | 1.06 |
| **T7.1.38** | 47.3 | 6.9* | 20.9 | 3.81 | 3.67 | 62.1 | 13.4 | 14.5 | 0.89 | 1.07 |
| **NC6.2** | 46.5 | 10.2 | 21.7 | 3.81 | 3.61 | 61.8 | 14.0 | 14.2 | 0.89 | 1.07 |
| **Null 7.6** | 46.8 | 8.2 | 21.6 | 3.81 | 3.65 | 61.3 | 14.1 | 14.7 | 0.93 | 1.08 |
| **WT** | 46.9 | 9.1 | 20.9 | 3.82 | 3.79 | 62.4 | 12.7 | 14.9 | 0.95 | 1.06 |
| SED | 0.59 | 0.52 | 0.29 | 0.04 | 0.09 | 0.55 | 0.62 | 0.33 | 0.02 | 0.01 |
| LSD (5%) | 1.20 | 1.05 | 0.72 | 0.08 | 0.18 | 1.10 | 1.2 | 0.67 | 0.05 | 0.03 |
| P Value | 0.006 | <0.001 | <0.001 | <0.001 | 0.058 | <0.001 | <0.001 | 0.001 | <0.001 | 0.173 |

SED, standard error of difference of means; LSD, least significant difference; NC, Null control; T, Transgenic; WT, wild type. * Values significantly different in transgenic line than corresponding null.
